# Inter-Conversion between Different Compounds of Ternary Cs-Pb-Br System

**DOI:** 10.3390/ma11050717

**Published:** 2018-05-02

**Authors:** Jing Li, Huijie Zhang, Song Wang, Debing Long, Mingkai Li, Duofa Wang, Tianjin Zhang

**Affiliations:** 1 Department of Materials Science and Engineering, Hubei University, Wuhan 430062, China; lijing5781@hotmail.com (J.L.); huijie928@163.com (H.Z.); debinglong@foxmail.com (D.L.); mingkailee@hotmail.com (M.L.); 2 Hubei Collaborative Innovation Center for Advanced Organic Chemical Materials, Hubei University, Wuhan 430062, China; 3 Hubei Key Laboratory of Low Dimensional Optoelectronic Materials and Devices, Hubei University of Arts and Science, Xiangyang 441053, China; wangsong1984@126.com; 4 Hubei Provincial Key Laboratory of Polymers, Hubei University, Wuhan 430062, China; 5 Ministry of Education Key Laboratory of Green Preparation and Application for Materials, Hubei University, Wuhan 430062, China

**Keywords:** CsPbBr_3_, Cs_4_PbBr_6_, CsPb_2_Br_5_, perovskite, conversion

## Abstract

The perovskite CsPbBr_3_ attracts great attention due to its potential in optoelectronics. However, stability remains a major obstacle to achieving its effecting application. In this work, we prepared CsPbBr_3_ solids through a simple reaction and investigated reversible conversion between CsPbBr_3_, Cs_4_PbBr_6_, and CsPb_2_Br_5_. We found that CsPbBr_3_ can be respectively converted to Cs_4_PbBr_6_ or CsPb_2_Br_5_ by reacting with CsBr or PbBr_2_. Thermodynamic analysis demonstrated that the chemical reactions above were exothermic and occurred spontaneously. Moreover, the formed Cs_4_PbBr_6_ could be converted to CsPbBr_3_ reversely, and then progressively converted to Cs-deficient CsPb_2_Br_5_ by extraction of CsBr with water. The CsPb_2_Br_5_ was converted to CsPbBr_3_ reversely under thermal annealing at 400 °C. The thermodynamic processes of these conversions between the three compounds above were clarified. Our findings regarding the conversions not only provide a new method for controlled synthesis of the ternary Cs-Pb-Br materials but also clarify the underlying mechanism for the instability of perovskites CsPbBr_3_.

## 1. Introduction

All-inorganic cesium lead halide perovskite CsPbX_3_ (X = I, Br, Cl) nanocrystals (NCs) have attracted considerable attention owing to the outstanding photophysical properties, such as high photoluminescence quantum yields, narrow emission bandwidths, and tunable band gaps that covers the full visible range [1,2]. Since the pioneering work by the Kovalenko group in 2015, considerable progress in the preparation and application of CsPbBr_3_ NCs has been achieved within a very short time period [1]. CsPbBr_3_ NCs with a controllable morphology and composition have been fabricated by different methods, such as hot-injection [3], solvothermal synthesis [4], room-temperature precipitation [5], and chemical vapor deposition (CVD) [6]. Moreover, a variety of photoelectronic devices—such as photovoltaics [7], lasing [8], light-emitting diodes and photodetectors [9,10]—have been prepared from CsPbBr_3_ NC. In addition to CsPbBr_3_ NCs, other types of materials of the ternary Cs-Pb-Br system, such as Cs_4_PbBr_6_ and CsPb_2_Br_5_, have also been reported [11,12]. The compounds CsPbBr_3_, Cs_4_PbBr_6_, and CsPb_2_Br_5_ differ in the stacking of PbBr_6_ octahedra in their crystal structures. In CsPbBr_3_, the lead halide octahedra share all corners and are electronically coupled in three directions in space. However, in the Cs_4_PbBr_6_ lattice the octahedra do not share any corners [11]. One lead atom and eight bromine atoms make up a hendecahedron with edge sharing in CsPb_2_Br_5_ [12]. It is reasonable to suspect that the existence of multiple compounds of Cs-Pb-Br system is probably relevant to the unstable luminescent property of CsPbBr_3_, which is the main obstacle on the progress of CsPbBr_3_. The photoluminescence quantum yield (PLQY) of colloidal CsPbBr_3_ NCs of ~90% decreases dramatically to below ~20% when they are in the solid phase (such as in a thin film). Different mechanisms have been proposed to explain the luminescence quenching, such as loss of the high quality of the NC by aggregation, removal of the surface passivation, and chemical decomposition of the materials [11,13]. Therefore, investigation on the inter-conversion between the Cs-Pb-Br compounds above is rather important.

Very recently, it has been reported that these ternary Cs-Pb-Br compounds can be inter-converted by physical and chemical treatments. Conversion of pre-synthesized CsPbBr_3_ NC to Cs_4_PbBr_6_ NCs have been reported by the extraction of PbBr_2_ through amine- and thiol-mediation method [14,15]. Furthermore, a reverse conversion from Cs_4_PbBr_6_ to CsPbBr_3_ has been reported by the Manna group through extraction of CsBr with Prussian Blue [16]. However, all the conversions above were performed on Cs-Pb-Br NCs with ligands on their surface and they were realized with mediation by a ligand. Investigations on the inter-conversion between the bare compounds of the Cs-Pb-Br system without ligand mediation are rarely reported, which is essential to reveal instability mechanism of CsPbBr_3_.

In this work, we prepared CsPbBr_3_ particles without ligands through a simple low temperature method and realized reversible conversions between CsPbBr_3_ and Cs_4_PbBr_6_, and between CsPbBr_3_ and CsPb_2_Br_5_. We combined our experimental observations with calculations of the total energy of the three Cs-Pb-Br compounds, we found that conversions of CsPbBr_3_ to Cs_4_PbBr_6_ and to CsPb_2_Br_5_ could take place spontaneously. However, the reverse conversions required external intervention. 

## 2. Materials and Methods 

### 2.1. Materials

Lead(II) bromide (PbBr_2_, Aladdin, 99.999%), cesium bromide (CsBr, Aladdin, 99.999%), hydrobromic acid (HBr, ≥40.0%), and *N*,*N*-dimethylformamide (DMF, Aladdin, 99.9%), were used without any further purification.

### 2.2. Synthesis of CsPbBr_3_

The synthesis of CsPbBr_3_ was performed via a simple reaction and crystallization method. Briefly, 0.5 mmol of PbBr_2_ and 0.5 mmol of CsBr were dissolved in 10 mL of DMF and stirred until completely dissolved. The mixture was then placed in an oven at 40 °C to evaporate the solvent and induce the reaction to produce CsPbBr_3_ solids.

### 2.3. Experiments on the Inter-Conversion between the Compounds

#### 2.3.1. Forward Conversion from CsPbBr_3_ to Cs_4_PbBr_6_ or CsPb_2_Br_5_

Cesium bromide (CsBr, 3 mmol) was first dissolved in hydrobromic acid (HBr, 2 mL). Pre-synthesized CsPbBr_3_ (1 mmol) was added to the solution and stirred to react with CsBr and produce Cs_4_PbBr_6_, which precipitated at the bottom of the mixture. The precipitate was collected by evaporating the HBr solvent. For the conversion to CsPb_2_Br_5_, lead(II) bromide (PbBr_2_, 1 mmol) was first dissolved in hydrobromic acid (HBr, 2 mL). Then the CsPbBr_3_ solid (1 mmol) was added to the solution with stirring to react with PbBr_2_. The CsPb_2_Br_5_ was produced and precipitated at the bottom of the mixture. The precipitate was collected by evaporating the HBr solvent. 

#### 2.3.2. Reverse Conversion from Cs_4_PbBr_6_ or CsPb_2_Br_5_ to CsPbBr_3_

A 0.25 mmol portion of Cs_4_PbBr_6_ was added to de-ionized water (1 mL) and stirred, to trigger the reverse conversion from Cs_4_PbBr_6_ to CsPbBr_3_. The conversion of CsPb_2_Br_5_ to CsPbBr_3_ was conducted by annealing the CsPb_2_Br_5_ solids for 4 h at 400 °C in air.

### 2.4. Materials Characterization

Crystal structures were measured with an X-ray diffractometer (Bruker D8 Advance, Karlsruhe, Baden-Wurttemberg, Germany) with Cu-Ka radiation (λ = 1.5406 Å). Scanning electron microscope (SEM) and energy dispersive spectrum (EDS) measurements were performed on a JSM7100F, Tokyo, Honshu, Japan. A transmission electron microscope (TEM) (FEI; Tecnai-G20 and 200 kV, Hillsboro, OR, USA) was used to characterize the microstructure of the CsPbBr_3_, Cs_4_PbBr_6_ and CsPb_2_Br_5_. Absorption spectra were measured on a UV-Visible-NIR spectrophotometer (SHIMADZU UV-3600, Kyoto, Honshu, Japan).

### 2.5. First-Principle Calculations

First-principle calculations were performed on the basis of density functional theory (DFT) as implemented in the QUANTUM ESPRESSO (QE) code. The exchange and correlation terms were described using the general gradient approximation (GGA) of Perdew-Burke-Ernzerhof (PBE). The energy cutoff for the plane wave basis set was 600 eV. The accuracy of the self-consistent field (SCF) energy convergence and the convergence accuracy of the internal stress of the crystal were less than 1.4 × 10^−5^ eV/atom and 0.05 Gpa, respectively. For the different alloy configurations, Monkhorst-Pack grids were determined automatically for the Brillouin zone integration and the KPPRA parameter was set to be 1000.

## 3. Results and Discussion

### 3.1. Synthesis of CsPbBr_3_ and Forward Conversion to Cs_4_PbBr_6_ and CsPb_2_Br_5_.

**Figure 1:**
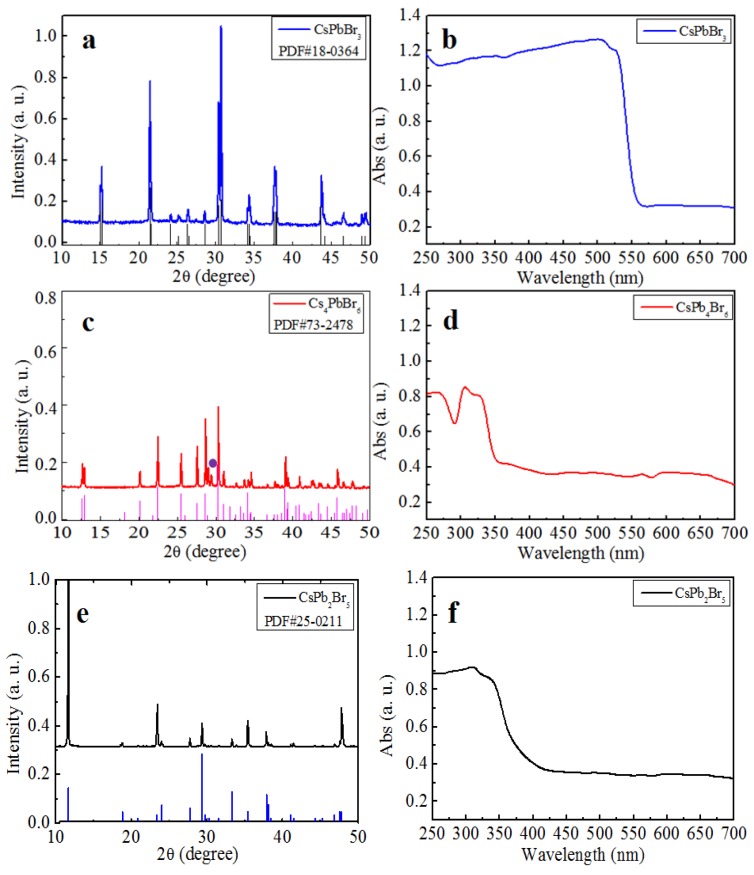
The XRD pattern of (**a**) CsPbBr_3_, (**c**) Cs_4_PbBr_6_ and (**e**) CsPb_2_Br_5_. The absorption spectra of (**b**) CsPbBr_3_, (**d**) Cs_4_PbBr_6_, and (**f**) CsPb_2_Br_5_.

Synthesis of CsPbBr_3_ (PDF#18-0364) was performed via a simple reaction and crystallization method without the use of any ligands. Full details are described in the experimental section. Characterization results of the prepared solids by XRD and absorption spectroscopy, shown in Figure 1a,b, demonstrated that the product was pure monoclinic CsPbBr_3_.

The as-synthesized CsPbBr_3_ solids were used to perform the forward conversion from CsPbBr_3_ to Cs_4_PbBr_6_ and CsPb_2_Br_5_. First, CsBr was dissolved in HBr, and a certain amount of the CsPbBr_3_ solid synthesized above (yellow) was added into the solution (CsPbBr_3_/CsBr = 1:3, mole ratio) with stirring. This approach ensured that only H was introduced into the reaction system, containing Cs, Pb, and Br, which simplified the analysis on the reaction. After stirring for several hours, a white precipitate formed, which was revealed to be rhombohedral Cs_4_PbBr_6_ by XRD, as shown in Figure 1c. To reveal whether CsPbBr_3_ remnants exist in the product since both CsPbBr_3_ and Cs_4_PbBr_6_ exhibit diffraction peaks near 27°, the absorption spectrum of Cs_4_PbBr_6_ product was measured and is shown in Figure 1d. A typical absorption peak at 315 nm was observed, which is characteristic of Cs_4_PbBr_6_, and no absorption peaks corresponding to CsPbBr_3_ appear [17]. This result further confirmed the conversion from CsPbBr_3_ to Cs_4_PbBr_6_. As far as the additional small diffraction peak near 29° denoted by purple dot in Figure 1c is concerned, it corresponds to CsBr. Because the ratio of the reactants CsPbBr_3_/CsBr was 1:3 and the only product was Cs_4_PbBr_6_, Equation (1) is proposed to describe the chemical reaction of the conversion. Except for operating as the solvent, the HBr also supplies abundance of Br^+^ and promotes the chemical reaction according to Equation (1). 

CsPbBr_3_ + 3CsBr = Cs_4_PbBr_6_ (1)

The conversion from CsPbBr_3_ to CsPb_2_Br_5_ was realized by a similar reaction. First, PbBr_2_ was dissolved in HBr and CsPbBr_3_ solid was added into the solution (CsPbBr_3_/PbBr_2_ = 1:1, mole ratio). After several hours, white solids precipitated at the bottom of the mixture. XRD characterization, as shown in Figure 1e, of the precipitate revealed that it was pure tetragonal CsPb_2_Br_5_, indicating that the conversion from CsPbBr_3_ to CsPb_2_Br_5_ occurred. To confirm the conversion, the absorption spectra of the reactant and product were measured. As shown in Figure 1f, the absorption edge moved to 380 nm, indicating that CsPbBr_3_ was converted into CsPb_2_Br_5_. The conversion was believed to occur through Equation (2), as shown below, based on the fact that the ratio of the reactants CsPbBr_3_/PbBr_2_ was 1:1 and the only product formed was CsPb_2_Br_5_.

CsPbBr_3_ + PbBr_2_ = CsPb_2_Br_5_ (2)

**Figure 2:**
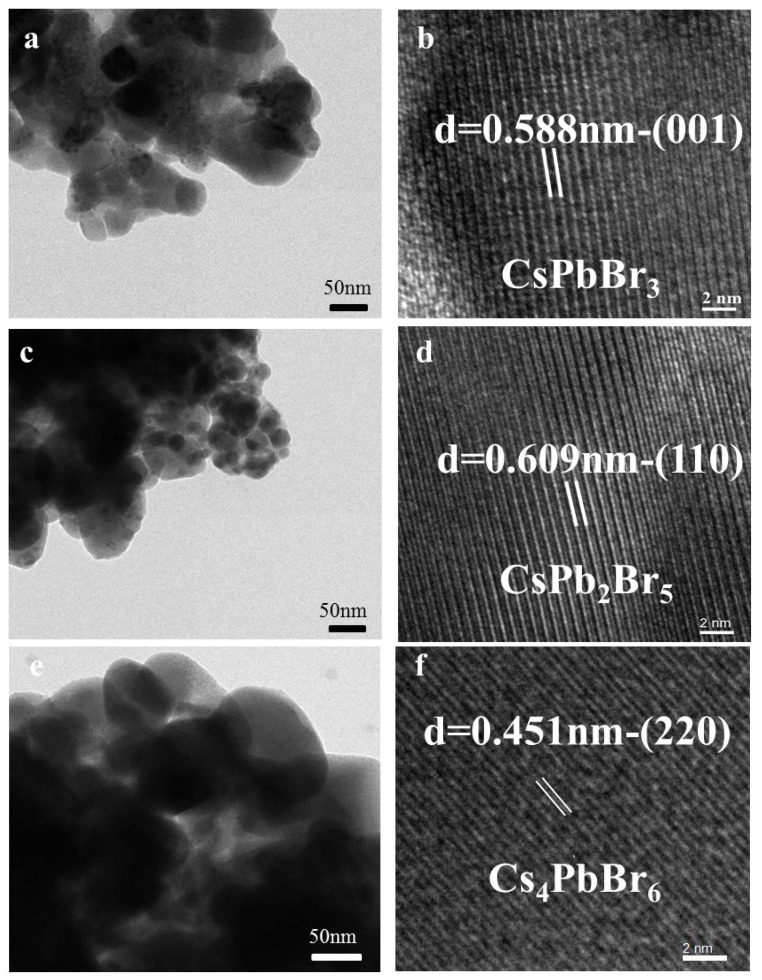
(**a**,**c**,**e**) TEM images; and (**b**,**d**,**f**) high-resolution lattice resolved TEM images of a representative CsPbBr_3_, CsPb_2_Br_5_, and Cs_4_PbBr_6_, respectively.

The morphology of each material was examined by scanning electron microscope (SEM) imaging, as shown in Figure S1. Energy dispersive spectroscopy (EDS) results, also shown in Figure S1, indicated that the molar ratios of Cs/Pb/Br were 1.18/1/2.89, 1/1.87/5.57, and 4.18/1/6.36 respectively, which agreed well with the stoichiometries of CsPbBr_3_, CsPb_2_Br_5_, and Cs_4_PbBr_6_. The microstructure of each Cs-Pb-Br ternary compound was characterized by high resolution TEM (HRTEM), as shown in Figure 2. Well-resolved lattice fringes were observed in the HRTEM images. In Figure 2b, the separation between the fringes was 0.588 nm, which corresponded to the (001) plane of CsPbBr_3_. In the HRTEM images of CsPb_2_Br_5_ and Cs_4_PbBr_6_, the (110) and (220) planes were clearly observed, with lattice separations of 0.609 and 0.451 nm, respectively. These EDS and HRTEM results further confirmed that the conversions had occurred.

In the conversions above, we did not use high temperature, high pressure or a catalyst to trigger the reactions. Hence, the Equations (1) and (2) are thermodynamically controlled process and the driving force, described by the free energy should be negative. Therefore, we calculated the total energy (Et) of the Equations (1) and (2) by first principles. The changes of the total energy (ΔEt) for Equations (1) and (2) were −9508.07 eV and −15019.13 eV, respectively, indicating that the chemical reactions were exothermic and could occur spontaneously. The total energy of each materials is shown in Table S1 in the supporting information. These results explain why this simple method can successfully realize the conversion of CsPbBr_3_ into Cs_4_PbBr_6_ or CsPb_2_Br_5_.

### 3.2. Reverse Conversion by Water Extraction and Thermal Annealing

For the reverse conversion from Cs_4_PbBr_6_ to CsPbBr_3_, we used the water extraction method proposed by the Sun group [18]. By mixing a Cs_4_PbBr_6_ quantum dot dispersion in nonpolar hexane with water, Sun et al. found that CsBr could be extracted from Cs_4_PbBr_6_ owing to the high solubility of CsBr in water. This effect led to a conversion from Cs_4_PbBr_6_ to CsPbBr_3_. 

Here, we found that water could also extract CsBr from Cs_4_PbBr_6_ solids without ligands on their surface. However, the reaction we observed was much more vigorous and quick. These differences between our observations and those of Sun et al. could be attributed to the absence of protective ligands on the surface of our NCs, unlike the Cs_4_PbBr_6_ quantum dot dispersion reported by Sun [18]. When the Cs_4_PbBr_6_ solids were added into deionized water, the color of the precipitate changed to yellow immediately, and returned to white again over a longer time, suggesting that chemical reactions occurred in two stages. The precipitate at different stages was removed from the deionized water and the composition was measured by XRD. 

**Figure 3:**
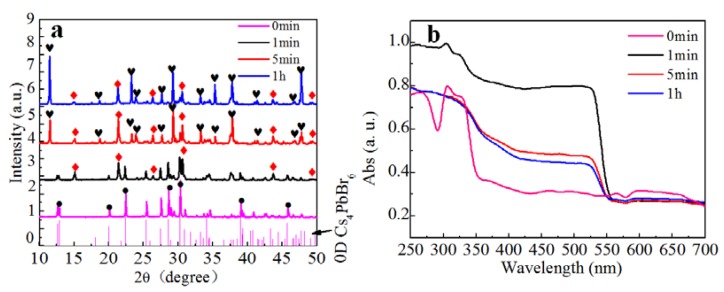
(**a**) PXRD pattern of Cs_4_PbBr_6_ solids after water treatment for different time, the red diamonds represent CsPbBr_3_, the black hearts represents CsPb_2_Br_5_, the black dots represents Cs_4_PbBr_6_; (**b**) Absorption spectra of PXRD pattern of Cs_4_PbBr_6_ solids after water treatment for different times.

As shown in Figure 3a, most of the Cs_4_PbBr_6_ transformed to CsPbBr_3_ within 1 min. However, CsPbBr_3_ was not the final product. The conversion into CsPb_2_Br_5_ proceeded within 5 min, and the major product was CsPb_2_Br_5_ after 1 h. Therefore, we suggest that the water not only extracted CsBr from Cs_4_PbBr_6_, but also extracted CsBr from CsPbBr_3_ to produce CsPb_2_Br_5_. The composition of the solution was also investigated to clarify the nature of the chemical transformation. To confirm the water extraction mechanism for the conversions, we evaporated the water and performed XRD measurements on the solid obtained, which was determined to be CsBr, as shown in the supporting information Figure S2. Therefore, we propose the following Equations for the chemical reactions occurring at each stage of the transformation as:

Cs_4_PbBr_6_ = CsPbBr_3_ + 3CsBr, (3)

2CsPbBr_3_ = CsPb_2_Br_5_ + CsBr (4)

Figure 3b shows the absorption spectrum measured from the precipitate samples removed from deionized water after different reaction times. A strong characteristic absorption edge at 560 nm appeared after 1 min and its intensity decreased at longer reaction times. This result indicates that the CsPbBr_3_ was produced within 1 min and converted to CsPb_2_Br_5_ over longer reaction times. These findings are consistent with the XRD results.

**Figure 4:**
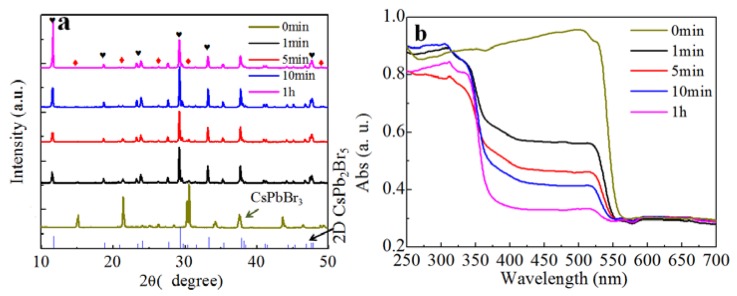
(**a**) XRD patterns and (**b**) absorption spectra of CsPbBr_3_ solids after water treatment for different times. Red diamonds represent CsPbBr_3_ and black hearts denote the diffraction peaks of CsPb_2_Br_5_.

To confirm the two-step transformation model suggested above, we added pure CsPbBr_3_ into deionized water and investigated the conversion. We found that the transformation in Equation (4) occurred and a white precipitate was formed quickly. As shown in the XRD pattern obtained from the precipitate in Figure 4a, the diffraction peaks related to CsPbBr_3_ became weak and strong diffraction peaks corresponding to CsPb_2_Br_5_ were observed after 1 min. Moreover, the intensity of CsPb_2_Br_5_ gradually increased as the reaction progressed. The absorption spectra in Figure 4b, show that the absorption peak at 560 nm from CsPbBr_3_ became progressively weaker. We note that the absorption peak of CsPbBr_3_ did not completely disappear, even after 1 h of reaction, indicating that a small amount of CsPbBr_3_ persisted. The XRD and absorption results clearly demonstrated that CsPbBr_3_ could be converted to CsPb_2_Br_5_ through extraction of CsBr by water. The conversion induced by water extraction is undoubtedly one of the reasons leading to the unstable luminescent property of CsPbBr_3_. The water vapor in the air can extract CsBr from CsPbBr_3_ and trigger the conversion into CsPb_2_Br_5_, which subsequently results in the degradation of luminescence.

**Figure 5:**
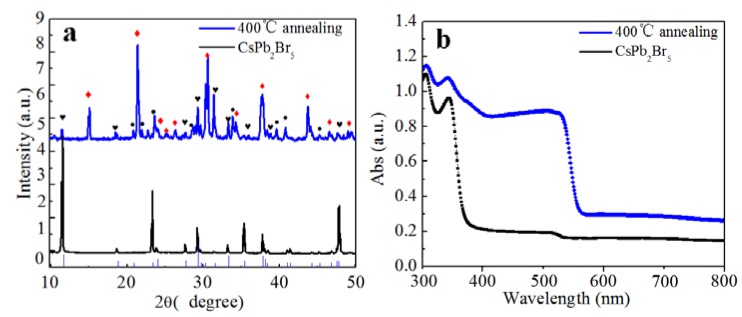
(**a**) XRD pattern, and (**b**) absorption spectra of CsPb_2_Br_5_ particles after annealing at 400 °C temperature. In the XRD pattern, the red diamonds denotes the diffraction peak of CsPbBr_3_, the black dots denotes the diffraction peak of PbBr_2_ and black hearts denote the diffraction peaks of CsPb_2_Br_5_.

We realized a conversion from CsPb_2_Br_5_ to CsPbBr_3_, using a previously reported annealing method [19]. We annealed the CsPb_2_Br_5_ solids at 400 °C in air for 4 h and monitored the associated XRD and absorption properties. Figure 5a shows XRD data of the CsPb_2_Br_5_ before and after annealing. The corresponding diffraction peaks before annealing were indexed to CsPb_2_Br_5_. After annealing, the main product corresponded to CsPbBr_3_ and PbBr_2_, and a small amount of CsPb_2_Br_5_ remained. The decomposition is depicted by the Equation:

CsPb_2_Br_5_ = CsPbBr_3_ + PbBr_2_ (5)

The absorption spectra in Figure 5b show that the absorption peak relevant to CsPbBr_3_ was considerably enhanced, indicating the generation of CsPbBr_3_, which is consistent with the XRD results shown in Figure 5a. As far as the mechanism of the Equation (5) is concerned, it is ascribed to the decompositon of CsPb_2_Br_5_ energetically driven by high temperature annealing. 

## 4. Conclusions

In conclusion, we examined a reversible conversion between CsPbBr_3_, Cs_4_PbBr_6_, and CsPb_2_Br_5_. First, CsPbBr_3_ solids were synthesized through a simple reaction of CsBr and PbBr_2_ in HBr. Addition of the prepared CsPbBr_3_ solids into the CsBr/PbBr_2_ solution in HBr, resulted in its conversion into Cs_4_PbBr_6_ and CsPb_2_Br_5_, through Equations (1) and (2), respectively. Thermodynamic analysis revealed that the transformations above were exothermic and occurred spontaneously. Moreover, we found that when added into the water Cs_4_PbBr_6_ converted to CsPbBr_3_ first, and then to Cs-deficient CsPb_2_Br_5_. These results are attributed to the ionic nature of the Cs-Pb-Br system and the high solubility of CsBr in water, which led to extraction of CsBr by water. The CsPb_2_Br_5_ was converted to CsPbBr_3_ through thermal annealing at 400 °C. Our results on the inter-conversion of the Cs-Pb-Br compounds sheds a light on understanding the mechanism and developing new solutions for the instability problem of the Cs-Pb-Br compounds. Moreover, it supplies important information on the controllable preparation of the Cs-Pb-Br materials.

## Supplementary Materials

The following are available online at http://www.mdpi.com/1996-1944/11/5/717/s1, Figure S1: Scanning electron microscope (SEM) and Energy dispersive X-spectroscopy (EDS); Figure S2: The XRD pattern of CsBr (PDF#73-0391) obtained from the solvent of water by evaporation; Table S1. The total energy of each materials is calculated based on the optimized structure using DFT.

## References

[1] Zhang J., Yang Y., Deng H., Farooq U., Yang X.K., Kan J., Tang J., Song H.S. (2017). High quantum yield blue emission from lead-free inorganic antimony halide perovskite colloidal quantum dot. ACS Nano.

[2] Protesescu L., Yakunin S., Bodnarchuk M.I., Krieg F., Caputo R., Hendon C.H., Yang R.X., Walsh A., Kovalenko M.V. (2015). Nanocrystals of cesium lead halide perovskites (CsPbX_3_, X = Cl, Br, and I): novel optoelectronic materials showing bright emission with wide color gamut. Nano Lett..

[3] Pan A., He B., Fan X., Liu Z., Urban J.J., Alivisatos A.P., He L., Liu Y. (2016). Insight into the ligand-mediated synthesis of colloidal CsPbBr_3_ perovskite nanocrystals: the role of organic acid, base, and cesium precursors. ACS Nano.

[4] Rakita Y., Kedem N., Gupta S., Sadhanala A., Kalchenko V., Böhm M.L., Kulbak M., Friend R.H., Cahen D., Hodes G. (2016). Low-temperature solution-grown CsPbBr_3_ single crystals and their characterization. Cryst. Growth Des..

[5] Sun S., Yuan D., Xu Y., Wang A., Deng Z. (2016). Ligand-mediated synthesis of shape-controlled cesium lead halide perovskite nanocrystals via reprecipitation process at room temperature. ACS Nano.

[6] Wang Y., Guan X., Li D., Cheng H.-C., Duan X., Lin Z., Duan X. (2017). Chemical vapor deposition growth of single-crystalline cesium lead halide microplatelets and heterostructures for optoelectronic applications. Nano Res..

[7] Swarnkar A., Marshall A.R., Sanehira E.M., Chernomordik B.D., Moore D.T., Christians J.A., Chakrabarti T., Luther J.M. (2016). Quantum dot–induced phase stabilization of α-CsPbI_3_ perovskite for high-efficiency photovoltaics. Science.

[8] Eaton S.W., Lai M., Gibson N.A., Wong A.B., Dou L., Ma J., Wang L.-W., Leone S.R., Yang P. (2016). Lasing in robust cesium lead halide perovskite nanowires. Proc. Natl. Acad. Sci. USA.

[9] Song J., Li J., Li X., Xu L., Dong Y., Zeng H. (2015). Quantum dot light-emitting diodes based on inorganic perovskite cesium lead halides (CsPbX_3_). Adv. Mater..

[10] Ramasamy P., Lim D.-H., Kim B., Lee S.-H., Lee M.-S., Lee J.-S. (2016). All-inorganic cesium lead halide perovskite nanocrystals for photodetector applications. Chem. Commun..

[11] Saidaminov M.I., Almutlaq J., Sarmah S., Dursun I., Zhumekenov A.A., Begum R., Pan J., Cho N., Mohammed O.F., Bakr O.M. (2016). Pure Cs_4_PbBr_6_: Highly luminescent zero-dimensional perovskite solids. ACS Energy Lett..

[12] Wang K.H., Wu L., Li L., Yao H.B., Qian H.S., Yu S.H. (2016). Large-scale synthesis of highly luminescent perovskite-related CsPb_2_Br_5_ nanoplatelets and their fast anion exchange. Angew. Chem. Int. Ed..

[13] Zhang Y., Saidaminov M.I., Dursun I., Yang H., Murali B., Alarousu E., Yengel E., Alshankiti B.A., Bakr O.M., Mohammed O.F. (2017). Zero-dimensional Cs_4_PbBr_6_ perovskite nanocrystals. J Phys. Chem. Lett..

[14] Liu Z., Bekenstein Y., Ye X., Nguyen S.C., Swabeck J., Zhang D., Lee S.-T., Yang P., Ma W., Alivisatos A.P. (2017). Ligand mediated transformation of cesium lead bromide perovskite nanocrystals to lead depleted Cs_4_PbBr_6_ nanocrystals. J Am. Chem. Soc..

[15] Palazon F., Almeida G., Akkerman Q.A., De Trizio L., Dang Z., Prato M., Manna L. (2017). Changing the dimensionality of cesium lead bromide nanocrystals by reversible postsynthesis transformations with Amines. Chem. Mater..

[16] Palazon F., Urso C., De Trizio L., Akkerman Q., Marras S., Locardi F., Nelli I., Ferretti M., Prato M., Manna L. (2017). Postsynthesis Transformation of insulating Cs_4_PbBr_6_ nanocrystals into bright perovskite CsPbBr_3_ through physical and chemical extraction of CsBr. ACS Energy Lett..

[17] Quan L.N., Quintero-Bermudez R., Voznyy O., Walters G., Jain A., Fan J.Z., Zheng X., Yang Z., Sargent E.H. (2017). Highly emissive green perovskite nanocrystals in a solid state crystalline matrix. Adv. Mater..

[18] Wu L., Hu H., Xu Y., Jiang S., Chen M., Zhong Q., Yang D., Liu Q., Zhao Y., Sun B. (2017). From nonluminescent Cs_4_PbX_6_ (X = Cl, Br, I) nanocrystals to highly luminescent CsPbX_3_ nanocrystals: water-triggered transformation through a CsX-stripping mechanism. Nano Lett..

[19] Li J., Zhang H., Wang S., Long D., Li M., Guo Y., Zhong Z., Wu K., Wang D., Zhang T. (2017). Synthesis of all-inorganic CsPb_2_Br_5_ perovskite and determination of its luminescence mechanism. RSC Adv..

