# Supplementary material for: Inter-Conversion between Different Compounds of Ternary Cs-Pb-Br System
Source: Materials (Basel). 2018 May 2;11(5):717. doi: 10.3390/ma11050717 (PMC5978094; doi:10.3390/ma11050717)
Supplement: Supplementary file 1 [file materials-11-00717-s001.pdf]

Supplementary Materials

Inter-conversion between Different Compounds of Ternary Cs-Pb-Br System

Jing Li [1], Huijie Zhang [1], Song Wang [2], Debing Long [1], Mingkai Li [1], Duofa Wang [1,*], and Tianjin Zhang [1,*]

[1] Hubei Collaborative Innovation Center for Advanced Organic Chemical Materials, Ministry of Education Key Laboratory of Green Preparation and Application for Materials, Hubei Provincial Key Laboratory of Polymers, Department of Materials Science and Engineering, Hubei University, Wuhan 430062, People's Republic of China; lijing5781@hotmail.com (J.L.); huijie928@163.com (H.Z.); debinglong@foxmail.com (D.L.); mingkailee@hotmail.com (M.L.)

[2] Hubei Key Laboratory of Low Dimensional Optoelectronic Materials and Devices, Hubei University of Arts and Science, Xiangyang, Hubei 441053, China; wangsong1984@126.com

* Correspondence: duofawang@hotmail.com (D.W.); Tel.: +86-27-8866-1729; zhangtj@hubu.edu.cn (T.Z.); Tel.: +86-27-8866-1729

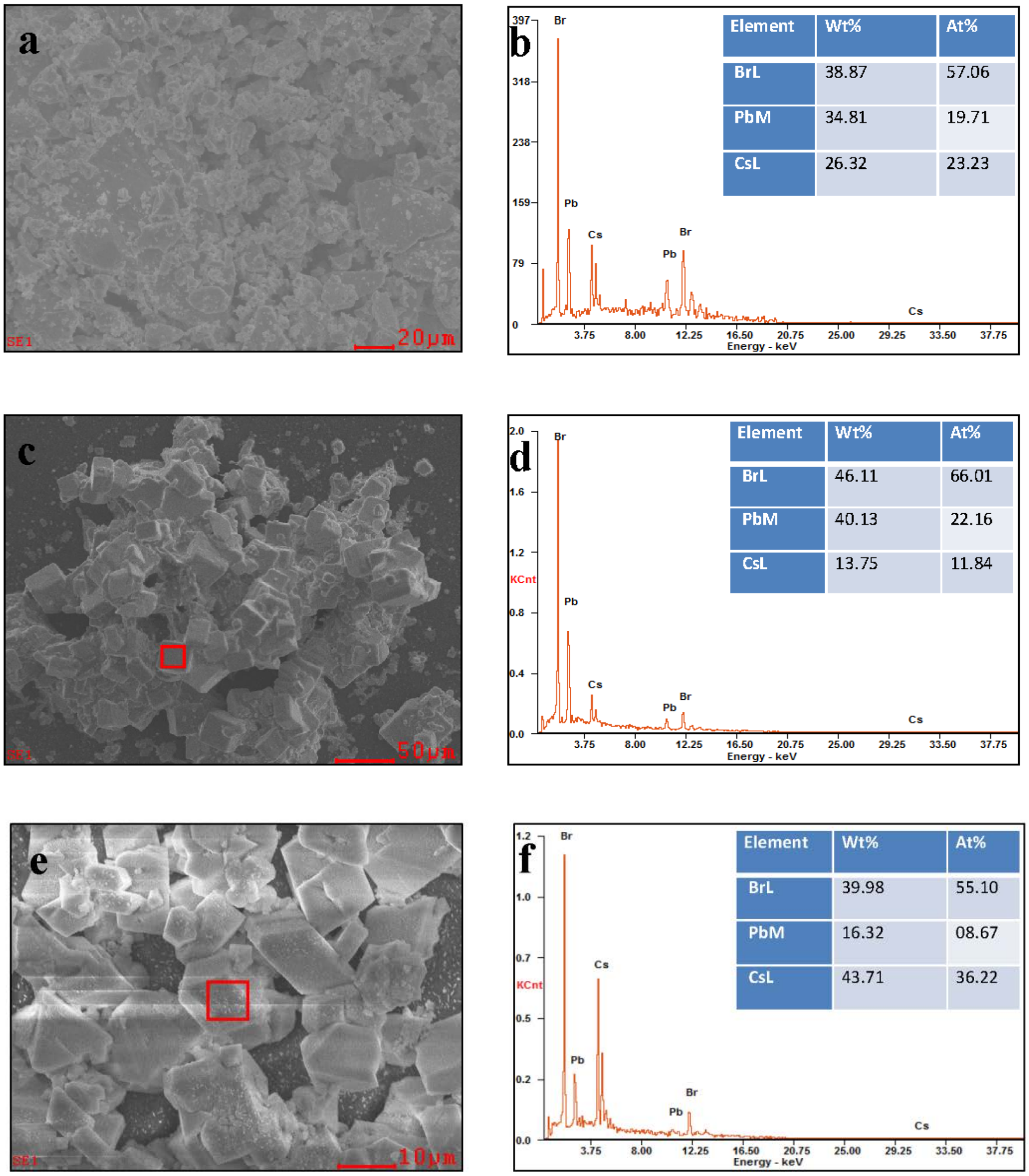

Figure S1. The SEM images of (**a**) $CsPbBr_3$, (**c**) Cs_4PbBr_6 and (**e**) $CsPb_2Br_5$. The Energy dispersive X-spectroscopy of (**b**) $CsPbBr_3$, (**d**) Cs_4PbBr_6 and (**f**) $CsPb_2Br_5$.

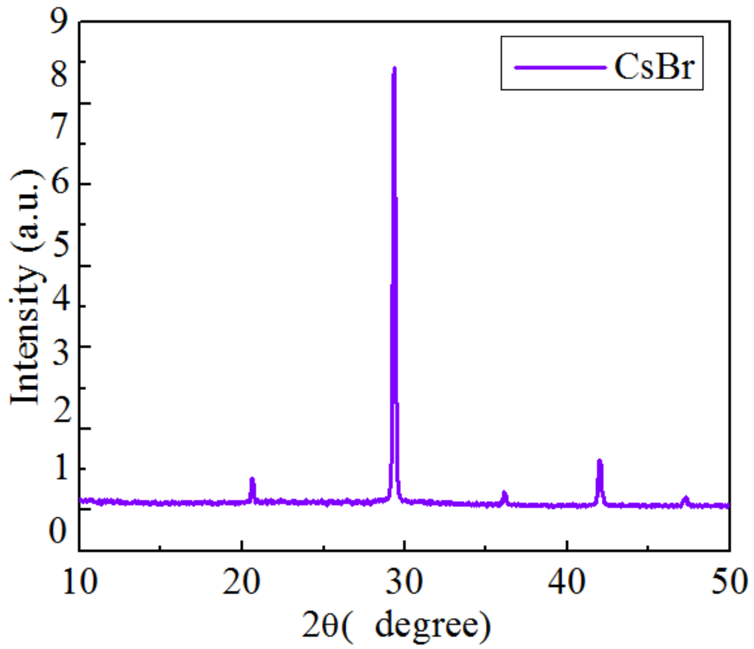

Figure S2. The XRD pattern of CsBr (PDF#73-0391) obtained from the solvent of water by evaporation.

Table S1. The total energy of each materials is calculated based on the optimized structure using DFT. The change of the total energy is obtained by the difference between the products and the reactants.

	$CsPbBr_3$	$PbBr_2$	$CsPb_2Br_5$	CsBr	Cs_4PbBr_6
**Total energy (Et)**	**-368.24550680 Ry**	**-1032.20505769 Ry**	**-2505.07266819 Ry**	**-110.17268316 Ry**	**-1397.59804554 Ry**

$$CsPbBr_3 + 3CsBr = Cs_4PbBr_6 \qquad (1)$$

$$\Delta Et = Et_{Cs4PbBr6} - Et_{CsPbBr3} - Et_{CsBr} = -698.83Ry = -9508.07\ eV$$

$$CsPbBr_3 + PbBr_2 = CsPb_2Br_5 \qquad (2)$$

$$\Delta Et = Et_{CsPb2Br5} - Et_{CsPbBr3} - 3Et_{PbBr2} = -1104.62\ Ry = -15029.13eV$$
